# Sex-specific associations between adipokine profiles and carotid-intima media thickness in the Cameron County Hispanic Cohort (CCHC)

**DOI:** 10.1186/s12933-023-01968-4

**Published:** 2023-08-31

**Authors:** Daeeun Kim, Aylin Memili, Hung-Hsin Chen, Heather M. Highland, Hannah G. Polikowsky, Mohammad Yaser Anwar, Susan T. Laing, Miryoung Lee, Joseph B. McCormick, Susan P. Fisher-Hoch, Jennifer E. Below, Kari E. North, Absalon D. Gutierrez

**Affiliations:** 1 Department of Epidemiology, Gillings School of Global Public Health, University of North Carolina at Chapel Hill, Chapel Hill, NC USA; 2 Department of Genetics, University of North Carolina at Chapel Hill, Chapel Hill, USA; 3 Vanderbilt Genetics Institute, Vanderbilt University Medical Center, Nashville, TN USA; 4 Department of Internal Medicine, The University of Texas Health Science Center at Houston, Houston, TX USA; 5 Department of Epidemiology, Human Genetics and Environmental Sciences, The University of Texas Health Science Center at Houston School of Public Health, Brownsville Regional Campus, Brownsville, TX USA; 6 Division of Endocrinology, Diabetes, and Metabolism, Department of Medicine, The University of Texas Health Science Center at Houston McGovern Medical School, Houston, TX USA

**Keywords:** Adipokines, Carotid intima-media thickness, Cardiometabolic health, Sex-differences, Population-based cohort, Mexican–American, Health disparities

## Abstract

**Background:**

Adipokines are hormones secreted from adipose tissue and are associated with cardiometabolic diseases (CMD). Functional differences between adipokines (leptin, adiponectin, and resistin) are known, but inconsistently reported associations with CMD and lack of studies in Hispanic populations are research gaps. We investigated the relationship between subclinical atherosclerosis and multiple adipokine measures.

**Methods:**

Cross-sectional data from the Cameron County Hispanic Cohort (N = 624; mean age = 50; Female = 70.8%) were utilized to assess associations between adipokines [continuous measures of adiponectin, leptin, resistin, leptin-to-adiponectin ratio (LAR), and adiponectin-resistin index (ARI)] and early atherosclerosis [carotid-intima media thickness (cIMT)]. We adjusted for sex, age, body mass index (BMI), smoking status, cytokines, fasting blood glucose levels, blood pressure, lipid levels, and medication usage in the fully adjusted linear regression model. We conducted sexes-combined and sex-stratified analyses to account for sex-specificity and additionally tested whether stratification of participants by their metabolic status (metabolically elevated risk for CMD as defined by having two or more of the following conditions: hypertension, dyslipidemia, insulin resistance, and inflammation vs. not) influenced the relationship between adipokines and cIMT.

**Results:**

In the fully adjusted analyses, adiponectin, leptin, and LAR displayed significant interaction by sex (p < 0.1). Male-specific associations were between cIMT and LAR [β(SE) = 0.060 (0.016), p = 2.52 × 10^–4^], and female-specific associations were between cIMT and adiponectin [β(SE) = 0.010 (0.005), p = 0.043] and ARI [β(SE) = − 0.011 (0.005), p = 0.036]. When stratified by metabolic health status, the male-specific positive association between LAR and cIMT was more evident among the metabolically healthy group [β(SE) = 0.127 (0.015), p = 4.70 × 10^–10^] (p for interaction by metabolic health < 0.1). However, the female-specific associations between adiponectin and cIMT and ARI and cIMT were observed only among the metabolically elevated risk group [β(SE) = 0.014 (0.005), p = 0.012 for adiponectin; β(SE) = − 0.015 (0.006), p = 0.013 for ARI; p for interaction by metabolic health < 0.1].

**Conclusion:**

Associations between adipokines and cIMT were sex-specific, and metabolic health status influenced the relationships between adipokines and cIMT. These heterogeneities by sex and metabolic health affirm the complex relationships between adipokines and atherosclerosis.

**Supplementary Information:**

The online version contains supplementary material available at 10.1186/s12933-023-01968-4.

## Background

Adipokines are hormones secreted from adipose tissue that have regulatory functions related to inflammatory and metabolic processes [1]. The molecular interactions of adipokines are susceptible to metabolic perturbations from chronic diseases such as obesity and cardiometabolic diseases (CMD), which suggests that adipokines may influence and be influenced by pathophysiological actions [2]. Among the diverse spectrum of adipokines, the roles of adiponectin and leptin are well-studied in the context of CMD development, but less so for resistin [3]. Generally, adiponectin protects against CMD risk as it is associated with anti-inflammatory pathways [4]. Leptin has been linked to both protective and proatherogenic states [5, 6], which is contingent on the integrity of leptin signaling. On the other hand, resistin has been more consistently implicated in cardiovascular disease (CVD) and pro-inflammatory states [7].

Among the plethora of CVD complications that have been associated with adipokine profile shifts, atherosclerosis, assessed using carotid intima-media thickness (cIMT), has been associated with decreased levels of adiponectin [8–10]. On the other hand, elevated leptin [11–13] and resistin [14–16] have been implicated in increased cIMT [14–16]. Emerging markers of adipose tissue function, leptin/adiponectin ratio (LAR) [or adiponectin/leptin ratio (ALR)] [17, 18] and adiponectin-resistin index (ARI) [19], have also been associated with cIMT [14, 20]. Thus, the role of adipokines in abnormal cIMT are still considerably inconsistent in the literature [8, 20–22].

Other notable research gaps include a lack of studies in Hispanic/Latino populations, inconsistent associations of adipokines and CMD by sex [23, 24], and inadequate consideration of pertinent CVD covariates [25], such as CMD related traits [26]. As there is heterogeneity in the clinical manifestation of CMD traits across racial groups and by sex, more population directed research is warranted.

In this study, we investigated the relationship between an early measure of subclinical atherosclerosis, cIMT, and adipokine levels, specifically adiponectin, leptin, and resistin, in a Hispanic/Latino cohort. We tested associations with two additional composite indices of adipose tissue function, LAR and ARI. We hypothesized that lower adiponectin levels would be associated with elevated cIMT, whereas higher leptin (and LAR), resistin (and ARI) would be associated with elevated cIMT in Hispanic/Latinos.

## Methods

### Study population

The Cameron County Hispanic Cohort (CCHC), initiated in 2004, is an ongoing community-based cohort study of Mexican Americans in Brownsville, TX. Households from the U.S. census tract/block were randomly ascertained through a two-stage sampling method. To date, more than 5000 individuals have been recruited, and demographic, lifestyle, and clinical factors were extensively examined by questionnaires, clinical examinations, and biospecimen collection. From the full cohort (N = 5020), we excluded participants from the pediatric study (age < 18 years; N = 392) and participants without exposure or outcome measures (N = 3815). We additionally excluded those who reported to have major cardiovascular events (i.e., heart attack and stroke) or a carotid endarterectomy (N = 26), were without genetic data (N = 24), whose adipokine levels or cytokine levels (as a covariate) were considered biologically implausible (as described in the following section) (N = 8), or missing covariates in primary analyses (N = 127). Ultimately, a total of 624 participants were included in the analysis. Adipokine typing began many years after the cohort began in 2004 thereby explaining the smaller sample size of this study (624 from a total cohort of 5020), yet distribution of age, sex, and BMI across participants in the sub-study were comparable to all CCHC participants. (Additional file 1: Table S3).

### Measures and definitions

#### Exposure

Circulating adipokines levels (explanatory variables)—adiponectin (ug/mL), leptin (ng/mL), and resistin (ng/mL)—and cytokines (covariates), including Interleukin-6 (IL-6; pg/mL), Interleukin-1 Beta (IL-1β; pg/mL), Interleukin-8 (IL-8; pg/mL), and Tumor Necrosis Factor-alpha (TNF-α; pg/mL), were assessed using the multiplex enzyme-linked immunosorbent assays (ELISA) (Milliplex Map, Millipore, CA) with two separate panels for different adipokines and cytokines [27]. Plasam samples were coated with analyte-specific antibody beads and analyzed using the Luminex 200 platform (Luminex Corp, Austin, TX) [27, 28]. We excluded abnormally high adiponectin measures (> 200 ug/mL) (N = 7) from our analysis as these values were biologically implausible and/or reflective of measurement errors in the assays. In addition, to avoid the biased estimation due to a few outliers, we winsorized the outliers of each adipokine (< 5% or > 95% of each adipokine measure). To carefully evaluate the potential influence of the outliers on the association between adipokines and cIMT, we performed multiple sensitivity analyses taking different approaches to treat the outliers (Additional file 1: Table S2). In addition to the three single adipokine measures (adiponectin, leptin, and resistin levels), two composite adipokine indices—LAR and ARI—were derived. LAR was calculated by dividing leptin levels (ng/mL) by adiponectin levels (ug/mL). ARI was calculated by applying a formulated index, [1 + log_10_(resistin levels (ng/mL)) − log_10_(adiponectin levels (ug/mL))], per previous derivations in the literature [19].

#### Outcome

Carotid ultrasound was conducted to assess subclinical atherosclerosis using the Siemens Acuson X300 ultrasound system (Malvern, PA) with a VF 13–5 linear array transducer, as previously described [29]. The implemented protocol aligned with the consensus statement on the application of carotid ultrasound for capturing subclinical vascular disease by the American Society of Echocardiography [30]. A total of 6 images of common arteries—i.e., anterior, lateral, and posterior images of left and right common carotid arteries—were taken. The cIMT measurements were ascertained with Carotid Analyzer software (Medical Imaging Applications, Coralville, IA), a semi-automated border detection program. Measurements were obtained at the R-wave of the electrocardiogram and on a minimum of 2 clips from each side. Continuous average cIMT measures in millimeters were recorded [30] and used in the current analysis.

#### Covariates

Measured weight (kg) and height (m) included in calculations of the body mass index (BMI; kg/m^2^) for participants. Information on smoking history (> 100 cigarettes in a lifetime) was collected. Fasting blood cytokine levels (IL-6, IL-1β, IL-8, and TNF-α) were measured through multiplex ELISA (Milliplex Map, Millipore, CA) along with adipokine levels. Glucose tolerance status was assessed with HbA1c and fasting blood glucose levels (FBG) (HbA1c < 5.7% and FBG < 100 mg/dL for normal), and normal or high blood pressure (BP) status was assessed via systolic blood pressure (SBP) and diastolic blood pressure (DBP) (SBP < 120 mmHg and DBP < 80 mmHg for normal BP). Lipid levels were stratified via high-density lipoprotein cholesterol (HDL-C), low-density lipoprotein cholesterol (LDL-C), and triglyceride (TG) levels. (HDL-C $\ge$ 40 mg/dL in males or $\ge$ 50 mg/dL in females, LDL-C < 160 mg/dL, and TG < 200 mg/dL, were considered normal). Type 2 Diabetes (T2D), hypertension, and lipid-lowering medication status were collected. In our post hoc investigation, participants were further classified into two groups based on their metabolic health status—metabolically elevated risk or metabolically healthy. As implemented in a previous study [29], the metabolically elevated risk group was defined as having at least two of the following components—(1) SBP ≥ 130 mmHg or DBP ≥ 85 mmHg or taking antihypertensive medication; (2) TG ≥ 150 mg/dL; (3) HDL < 40 mg/dL in males or < 50 mg/dL in females; (4) FBG ≥ 100 mg/dL or taking hypoglycemic medication; (5) Homeostatic Model Assessment for Insulin Resistance (HOMA-IR) > 5.13; or (6) C-reactive protein (CRP) > 3 mg/L. Multiple imputations by chained equations were conducted to impute missing CRP measures (N = 308) and HOMA-IR measures (N = 9) based on all the measured variables included in the fully adjusted model using Predictive Mean Matching approach in *mice* R package (10 imputations were performed).

#### Kinship matrix

We accounted for the genetic relatedness structure in CCHC participants by adjusting for a familial relationship (calculated as a kinship matrix) when estimating the association between adipokine levels and cIMT. We leveraged participants’ genome-wide genotype information to generate a kinship matrix using the *GENESIS* R package. Participants were genotyped on the MEGA-EX Array panel at the Vanderbilt University Medical Center Genotyping core facility, VANTAGE and undergone standard quality control processes (i.e., filtering the variants or samples with variant call rate < 90%, minor allele frequency < 0.01, sample or variant missing rate > 5%, deviation from Hardy–Weinberg Equilibrium (p < 1 × 10^−10^) as well as checks for heterozygosity and sex). A total of 182,799 single nucleotide polymorphisms were used in generating the kinship matrix.

### Statistical analysis

Descriptive summary statistics of variables were calculated as a mean and a standard deviation for continuous variables and the count and percentage for categorical variables using SAS 9.4 (SAS Institute Inc., Cary, NC). We performed a linear mixed effects model to test the association between continuous cIMT measures and adipokines, while accounting for the relatedness among CCHC participants with the kinship matrix included as a random effect. Explanatory variables were three single adipokine levels (adiponectin, leptin, and resistin) and two composite adipokine indices (LAR and ARI), and each explanatory variable was included in each regression model separately. Other covariates, described below, were included as fixed effects in the linear mixed-effect model. The linear mixed-effect model was implemented using the *lme4qtl* package in R.

We ran linear mixed-effect regression models and assessed covariate effects by adding up the group of covariates as followed: (1) demographic variables (age and sex), (2) lifestyle variables (BMI and smoking status), (3) cytokines (IL-1β, IL-6, TNF-α, and IL-8), (4) metabolic profile (normal vs. elevated risk in glycemic, blood pressure, lipid profile), and (5) medication status (T2D, hypertension, and lipid-lowering medication). Thus, the covariates in each model are age, sex in Model 1; age, sex, BMI, and ever smoking in Model 2; age, sex, BMI, ever smoking, and cytokines (IL-6, IL-8, TNF-a, IL-1b) in Model 3; age, sex, BMI, ever smoking, cytokines, and metabolic health (glucose tolerance, BP, and lipid profile) in Model 4; age, sex, BMI, ever-smoking, cytokines, metabolic health, and medication usage for T2D, hypertension, or lipid-lowering in Model 5 (the fully adjusted model). We thereby demonstrate how the estimated associations between adipokine levels and cIMT measures were influenced by different categories of covariates and present the effect estimates from all five models.

Adipokine levels were standardized to a mean of 0 and a standard deviation (SD) of 1. Thus, beta coefficients for regression analyses for the continuous cIMT measures were interpreted as the estimated increase in cIMT levels per 1-SD increase of adipokine or adipokine index levels.

In our primary analyses, we conducted both sexes-combined and sex-stratified analyses. We tested the interaction by sex at the significance level of p < 0.1. In our post hoc investigation, we further stratified the participants by their metabolic health status (metabolically elevated risk or not) and conducted regression analyses within strata to evaluate the potential influences of metabolic health status on the interplay between adipokines and cIMT. Interaction by metabolic health status was also tested.

## Results

### Characteristics of study participants

A total of 624 participants (442 females; 182 males) were included in this cross-sectional study. Descriptive statistics of study participants are summarized in Table 1. Of note, the mean BMI of participants was notably higher [30.7 kg/m^2^ (SD: 6.03)] than the average BMI of adults in the US [31], and about 20% of the overall participants reported taking a medication for either T2D, hypertension, or lipid-lowering purposes. Also, in this subset of CCHC subjects, more than 80% of the participants were classified as having metabolically elevated risk for CMD. Females had significantly (p < 0.05) higher levels of adiponectin, leptin, LAR, and IL-1β, lower levels of ARI, higher proportion of normal blood pressure profile, lower proportion of life-time smokers and normal lipid profile, and were more often taking T2D medications, as compared to males. When comparing cIMT levels, males had higher average cIMT measurements (0.71 ± 0.19 mm), as compared to females (0.66 ± 0.14 mm) (p < 0.05).

**Table 1:** Distributions of variables among the Cameron County Hispanic Cohort (CCHC) study participants

	Combined (N = 624)	Male (N = 182)	Female (N = 442)	p-values^e^
	Mean or N	Mean or N	Mean or N	
Adipokines				
Adiponectin (ug/mL)	26.3 (21.5)	19.5 (18.3)	29.1 (22.1)	< 0.001
Leptin (ng/mL)	21.9 (15.3)	11.0 (10.3)	26.4 (14.8)	< 0.001
Resistin (ng/mL)	34.5 (16.5)	35.0 (17.5)	34.3 (16.1)	0.661
Leptin-adiponectin ratio (LAR)	1.24 (1.15)	0.82 (0.87)	1.41 (1.21)	< 0.001
Adiponectin-Resitin Index (ARI)	1.18 (0.31)	1.31 (0.30)	1.12 (0.29)	< 0.001
cIMT				
Average cIMT (mm)	0.67 (0.15)	0.71 (0.19)	0.66 (0.14)	0.003
Potential confounders				
Age (years)	50.2 (14.4)	48.6 (15.7)	50.8 (13.7)	0.097
BMI (kg/m^2^)	30.7 (6.03)	30.2 (5.94)	30.9 (6.05)	0.174
Life time smoking > 100 cigarettes (N, %)	182 (29.2%)	99 (54.4%)	83 (18.8%)	< 0.001
Cytokines				
InterLeukin-6 (pg/mL)	4.91 (6.77)	4.55 (5.25)	5.06 (7.31)	0.331
InterLeukin-1 Beta (pg/mL)	1.83 (6.55)	1.20 (1.26)	2.08 (7.73)	0.02
InterLeukin-8 (pg/mL)	9.14 (8.70)	9.69 (7.91)	8.91 (9.00)	0.284
Tumor Necrosis Factor alpha (pg/mL)	5.27 (3.58)	5.49 (3.65)	5.17 (3.55)	0.311
Metabolic profile				
Normal glucose tolerance (yes) (N, %)^a^	215 (34.5%)	56 (30.8%)	159 (36.0%)	0.25
Normal blood pressure (N, %)^b^	342 (54.8%)	86 (47.3%)	256 (57.9%)	0.019
Normal lipid level (N, %)^c^	239 (38.3%)	87 (47.8%)	152 (34.4%)	0.002
Medications				
T2D Medication (yes) (N, %)	123 (19.7%)	26 (14.3%)	97 (21.9%)	0.038
Hypertension medication (yes) (N, %)	195 (31.3%)	55 (30.2%)	140 (31.7%)	0.794
Lipid lowering medication (yes) (N, %)	172 (27.6%)	47 (25.8%)	125 (28.3%)	0.599
Metabolic health				
Metabolically elevated risk^d^ (yes) (N, %)	507 (81.3%)	146 (80.2%)	361 (81.7%)	0.756

^a^Normal glucose tolerance: HbA1c < 5.7 and fasting blood glucose < 100

^b^Normal blood pressure: SBP < 120 and DBP < 80

^c^Normal lipid levels: HDL-C ≥ 40 for males and ≥ 50 for females, LDL-C < 160, and TG < 200

^d^Metabolically elevated risk defined as having at least 2 of the following components: (1) SBP ≥ 130 mmHg or DBP ≥ 85 mmHg or taking antihypertensive medication; (2) TG ≥ 150 mg/dL; (3) HDL < 40 mg/dL in males or < 50 mg/dL in females; (4) FBG ≥ 100 mg/dL or taking hypoglycemic medication; (5) HOMA-IR > 5.13; (6) CRP > 3 mg/L

^e^p-values from t-test (for continuous variables) and chi-square test (for binary variables) for the sex-differences

### Key associations between adipokines and cIMT

**Fig. 1:**
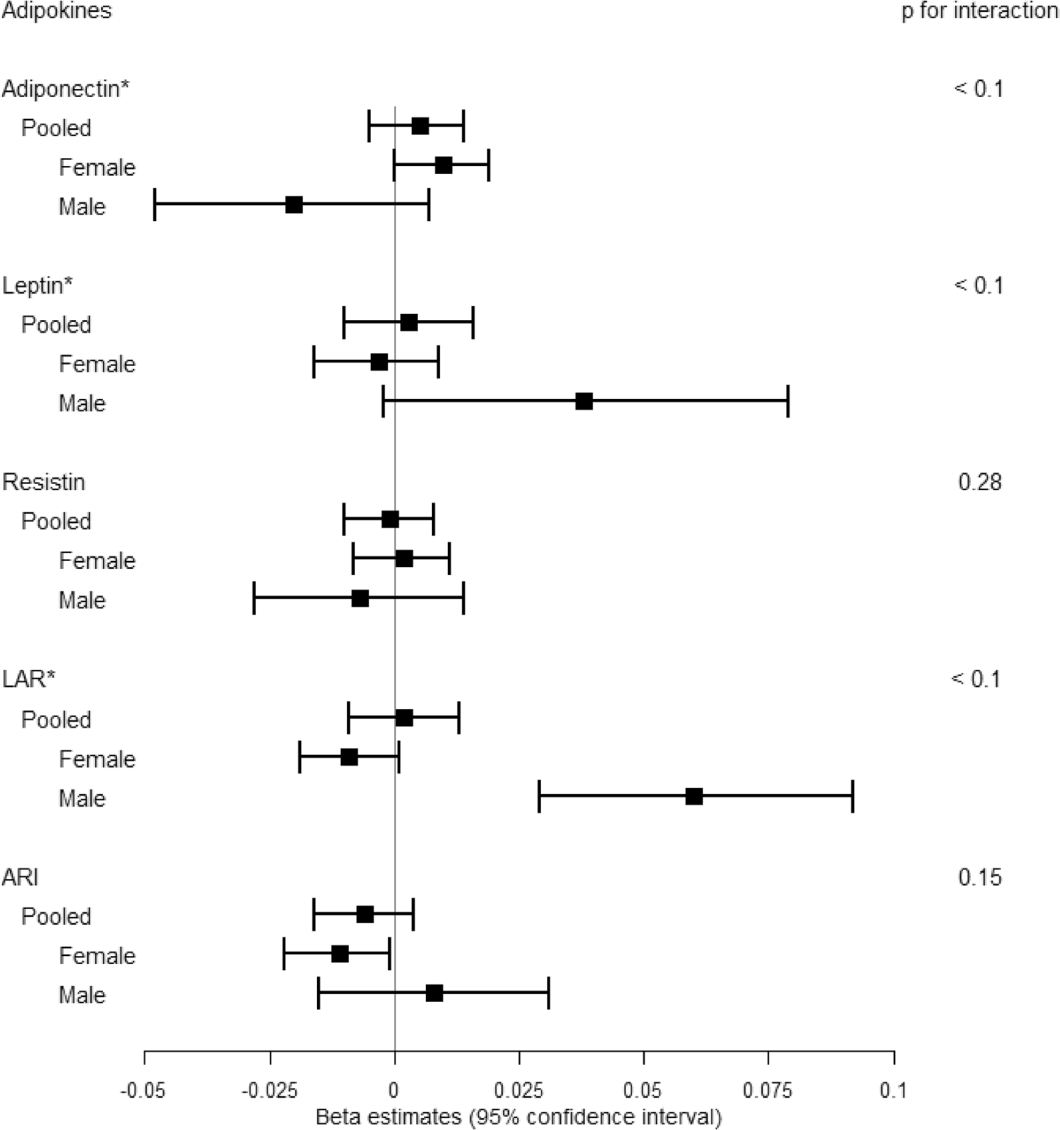
**Associations between adipokines and cIMT in both sexes-combined, females, and males.** Associations between adipokines and cIMT (continuous) from the fully adjusted linear mixed effects model (Model 5) are demonstrated on the forest plot. Beta coefficients and 95% confidence interval indicate the increase of cIMT levels by every 1-SD increase of adipokines [1-SD: 21.5 ug/mL for adiponectin, 15.3 ng/mL for leptin, 16.5 ng/mL for resistin, 1.15 for LAR, 0.31 for ARI]. Results for sexes-combined and sex-stratified analyses are shown. Significant interaction by sex (p for interaction < 0.1) is indicated (*)

Associations of adipokine measures with continuous cIMT measures across each adjustment to the linear regression models are reported in Table 2. In the sexes-combined fully adjusted model, no significant associations between any adipokine and cIMT measurements were observed. However, associations between adipokine and cIMT demonstrated significant interactions by sex (p < 0.1) for adiponectin, leptin and LAR (Fig. 1 and Table 2).

**Table 2:** Estimated association between adipokines and cIMT (continuous) in CCHC study participants

	Model 1*			Model 2 *			Model 3*			Model 4*			Model 5*		
	Beta	SE	p	Beta	SE	p	Beta	SE	p	Beta	SE	p	Beta	SE	p
Both sexes-combined															
Adiponectin^a^	0.004	0.005	0.472	0.005	0.005	0.358	0.002	0.005	0.716	0.003	0.005	0.520	0.005	0.005	0.350
Leptin^a^	0.004	0.005	0.427	0.003	0.007	0.680	− 0.001	0.007	0.860	0.001	0.007	0.930	0.003	0.007	0.655
Resistin	0.003	0.005	0.568	0.002	0.005	0.634	0.000	0.005	0.954	− 0.001	0.005	0.839	− 0.001	0.005	0.810
LAR^a^	0.004	0.005	0.423	0.003	0.006	0.642	0.002	0.006	0.713	0.002	0.006	0.723	0.002	0.006	0.730
ARI	− 0.001	0.005	0.867	− 0.002	0.005	0.658	− 0.002	0.005	0.700	− 0.004	0.005	0.415	− 0.006	0.005	0.243
Female															
Adiponectin	0.008	0.005	0.074	0.009	0.005	0.060	0.007	0.005	0.170	0.008	0.005	0.076	0.010	0.005	0.043
Leptin	− 0.002	0.005	0.741	− 0.003	0.006	0.634	− 0.007	0.006	0.291	− 0.005	0.006	0.436	− 0.003	0.006	0.588
Resistin	0.006	0.005	0.263	0.005	0.005	0.330	0.002	0.005	0.631	0.002	0.005	0.713	0.002	0.005	0.754
LAR	− 0.006	0.005	0.184	− 0.008	0.005	0.130	− 0.009	0.005	0.089	− 0.009	0.005	0.075	− 0.009	0.005	0.069
ARI	− 0.005	0.005	0.351	− 0.006	0.005	0.270	− 0.006	0.005	0.244	− 0.009	0.005	0.083	− 0.011	0.005	0.036
Male															
Adiponectin	− 0.016	0.013	0.240	− 0.015	0.013	0.244	− 0.020	0.014	0.146	− 0.020	0.014	0.154	− 0.020	0.014	0.148
Leptin	0.043	0.016	0.011	0.041	0.020	0.048	0.036	0.021	0.087	0.038	0.021	0.070	0.038	0.021	0.064
Resistin	− 0.001	0.011	0.898	− 0.002	0.011	0.859	− 0.006	0.011	0.575	− 0.007	0.011	0.546	− 0.007	0.011	0.521
LAR	0.060	0.014	4.41E−05	0.062	0.016	1.39E−04	0.061	0.016	2.12E−04	0.062	0.016	1.90E−04	0.060	0.016	2.52E−04
ARI	0.010	0.011	0.397	0.009	0.011	0.441	0.010	0.011	0.396	0.009	0.012	0.451	0.008	0.012	0.477

^*^Linear mixed effect model was applied. A kinship matrix estimated based on genotype information among samples was included as a random effect. Fixed effect covariates were age, sex in model 1; age, sex, BMI, and ever smoking in model 2; age, sex, BMI, ever smoking, and cytokines (IL-6, IL-8, TNF-a, IL-1b) in model 3; age, sex, BMI, ever smoking, cytokines, and metabolic health (glucose tolerance, BP, and lipid profile) in model 4; age, sex, BMI, ever-smoking, cytokines, metabolic health, and medication usage for T2D, hypertension, or lipid-lowering in model 5. (Sex was included only in the sexes-combined models)

Beta coefficients indicate the increase in cIMT levels by every 1-SD unit increase of adipokines

^a^Significant interaction by sex (p for interaction < 0.1) from Model 5

When stratified by sex, higher adiponectin levels and lower ARI were associated with increased cIMT among females [β(SE) = 0.010 (0.005), p = 0.043 for adiponectin; β(SE) = − 0.011 (0.005), p = 0.036 for ARI] in the fully adjusted Model 5. The same variables (in same model) displayed opposite directions of effect in males but were statistically nonsignificant. Among males, one significant association was observed—higher LAR was associated with increased cIMT across all adjustment models [e.g., β(SE) = 0.060 (0.016), p = 2.52 × 10^–4^ in Model 5]. The effect estimate for LAR (in the same model) moved in the opposing direction among females without statistical significance (Fig. 1 and Table 2).

### Testing the influence of metabolic health status on the associations between adipokines and cIMT

**Fig. 2:**
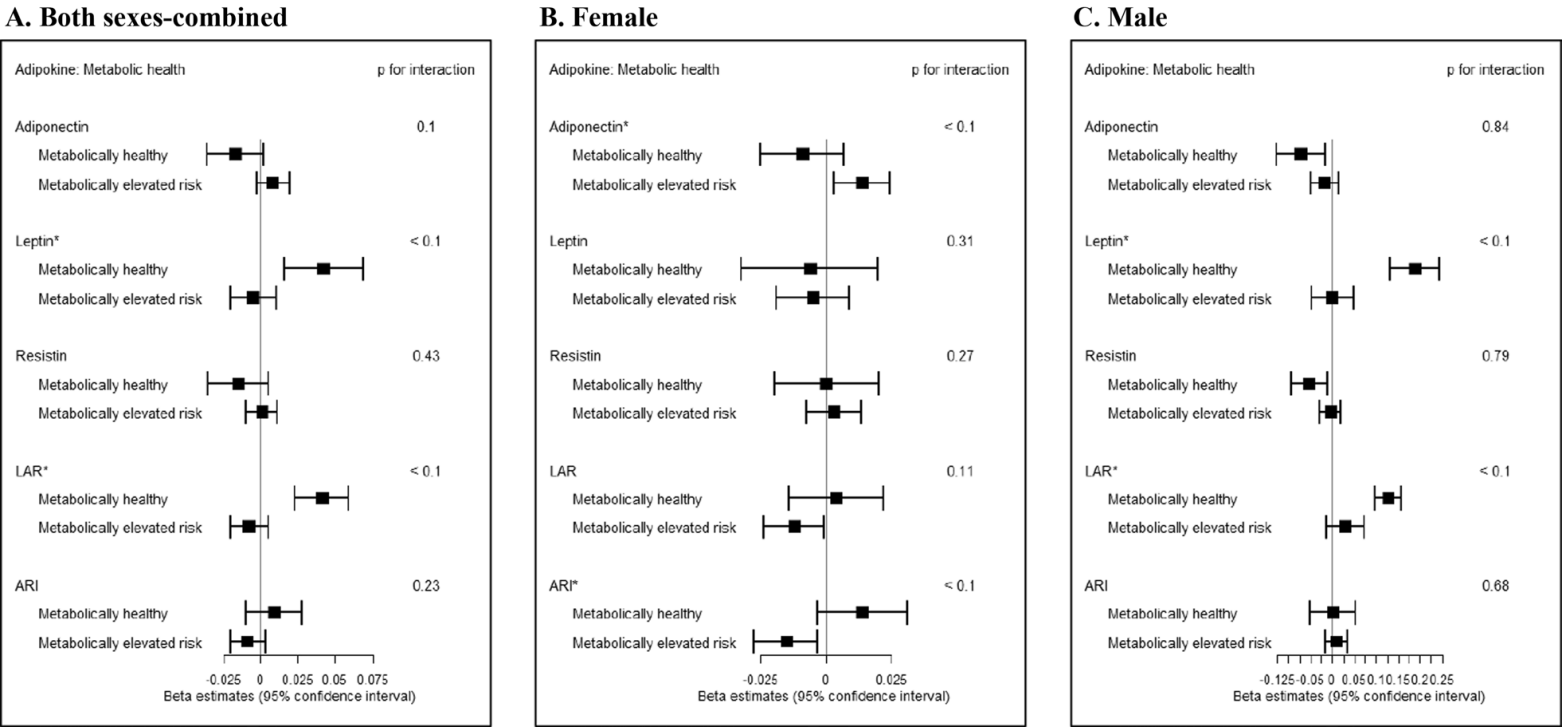
**Associations between adipokines and cIMT stratified by metabolic health status.** Associations between adipokines and cIMT levels stratified by metabolic health status from the fully adjusted linear mixed effects model are demonstrated on the forest plot (A. Both sexes-combined, B. Female, and C. Male) Beta coefficients (95% confidence intervals) indicate the increase in cIMT levels by every 1-SD increase of adipokines [1-SD: 21.5 ug/mL for adiponectin, 15.3 ng/mL for leptin, 16.5 ng/mL for resistin, 1.15 for LAR, 0.31 for ARI]. Significant interaction by metabolic health status (p for interaction < 0.1) is indicated by * on the name of adipokines

Figure 2 and Additional file 1: Table S1 summarize the effect estimates from the fully adjusted regression model (Model 5 from Table 2) of adipokines and cIMT when participants are stratified by metabolic health status. In contrast to the metabolically elevated risk group, the metabolically healthy group showed positive associations of leptin and LAR with cIMT [β(SE) = 0.042 (0.014), p = 0.002 for leptin; β(SE) = 0.041 (0.009), p = 4.86 × 10^–5^ for LAR] in the sexes-combined analyses, which was not observed in the pooled (metabolically healthy and elevated risk groups combined) analyses (p for interaction by metabolic health status < 0.1 for leptin and LAR).

In the sex-specific analyses, a positive association between adiponectin and cIMT and an inverse association between ARI and cIMT were observed only among females in the metabolically elevated risk group [β(SE) = 0.014 (0.005), p = 0.012 for adiponectin; β(SE) = − 0.015 (0.006), p = 0.013 for ARI]. In addition, an inverse association between LAR and cIMT was observed among metabolically elevated risk group (β(SE) = − 0.012 (0.006), p = 0.040) among females. Among metabolically healthy females, the estimated associations for adiponectin, ARI, and LAR were in opposing directions, though not significant [β(SE) = − 0.009 (0.008), p = 0.267 for adiponectin; β(SE) = 0.014 (0.009), p = 0.113 for ARI; β(SE) = 0.004 (0.009), p = 0.690 for LAR], and the interactions by metabolic health status were significant (p < 0.1) for adiponectin and ARI among females.

Among males, four adipokine measures (adiponectin, leptin, resistin, and LAR) demonstrated significant associations with cIMT only in the metabolically healthy group. Lower adiponectin levels and resistin levels and higher leptin levels and LAR were associated with increased cIMT measures (β(SE) = − 0.072 (0.028), p = 0.015 for adiponectin; β(SE) = 0.188 (0.029), p = 1.04 × 10^–7^ for leptin; β(SE) = − 0.053 (0.021), p = 0.017 for resistin; and β(SE) = 0.127 (0.015), p = 4.70 × 10^–10^ for LAR) among males in the metabolically healthy group. The interactions by metabolic health were significant for leptin and LAR (p < 0.1).

## Discussion

In this cross-sectional study of Mexican Americans in the CCHC cohort, we investigated the association of adiponectin, leptin, resistin, and composite measures of ARI and LAR with an early marker of atherosclerosis, cIMT, both across and within sexes. Based on previous literature, we expected inverse associations of adiponectin [3] and positive associations of resistin (and ARI) [19, 32] as well as leptin (and LAR) [18, 33] with cIMT. We also hypothesized sex-specificity in these associations to a certain degree, as adipokine levels show variation by sex [34]. We observed sexual dimorphisms, measured as significant interaction by sex, in associations between adipokines and cIMT for adiponectin, leptin, and LAR. Specifically, we observed the hypothesized positive associations between LAR and cIMT among males and unexpected positive associations for adiponectin and inverse associations for ARI among females. We also observed the heterogeneities of these sex-specific associations by metabolic health status. The male-specific association for LAR was specific to the metabolically healthy group, whereas the female-specific associations for adiponectin or ARI were specific to the metabolically elevated risk group.

First, we observed the expected positive direction of effect in the association of a composite index of leptin and adiponectin, LAR, with cIMT among males across all adjustment models. The associations between LAR and cIMT, as well as leptin and cIMT, were further strengthened by restricting analyses to males in the metabolically healthy group and remained significant even among both sexes-combined in the metabolically healthy group (Additional file 1: Table S1). This finding aligns with previous studies of the LAR (or ALR) with CVD-related traits [35–43], including insulin resistance [39], metabolic perturbations [44], and T2D [45]. Although the directions of associations for single measure of adiponectin or leptin were consistent with previous literature among males and among the both sexes-combined metabolically healthy group, the associations were weaker compared to analyses with LAR (Additional file 1: Table S1). Studies applying LAR have indicated an improvement of CVD risk prediction by applying the composite index compared to assessing adiponectin and/or leptin measures alone [46]. Thus, our findings support the notion that composite indices of adipokines expand our understanding of adipose tissue dysfunction [17, 47–51] and suggest that LAR may serve as a better surrogate biomarker for early atherosclerosis among males or among metabolically healthy group than single adipokine measures. However, while LAR may be a promising metric, our data also support the importance of sex-specific differences in the sensitivity and specificity of LAR for assessing metabolic health [52]. Furthermore, given that single leptin measures were more strongly associated with cIMT compared to single adiponectin measures, the significant relationship between LAR and cIMT was largely driven by leptin instead of adiponectin (Additional file 1: Table S1 and Table 2).

By measuring effect modification by sex in the associations between adipokines and cIMT, we further elucidated sex-specific relationships. We identified significant effect modification by sex (p for interaction by sex < 0.1) (Table 2 and Fig. 1), with opposing directions of association in females and males for adiponectin, leptin, and LAR measures. Among females, higher adiponectin levels (with statistical significance), lower leptin levels (without statistical significance), and lower LAR levels (without statistical significance) were associated with higher levels of cIMT; whereas among males, lower adiponectin levels (without statistical significance), higher leptin levels (without statistical significance), and higher LAR levels (with statistical significance) were associated with higher cIMT measures, in line with the previous literature. By restricting participants to the metabolically healthy group, sex-specific analyses indicated heterogeneous associations between adipokines and cIMT across sexes – i.e., metabolically healthy males showed more evident associations consistent with previous literature, except for resistin, but no remarkable associations were observed among metabolically healthy females. Instead, females-only of the metabolically elevated risk group displayed multiple significant, but counterintuitive, associations of adiponectin, LAR, and ARI with cIMT.

Sexual dimorphism in adipokine biology is well-known [53] and is closely related to sex-differences in body composition [54]. Hormonal differences emerge in puberty when sex steroid hormones cause distinct effects on body-fat distribution in males and females—i.e., females typically have greater fat mass than males, whereas males typically have greater lean mass and mineral mass than females [54]. Since adipokines are primarily secreted from adipose tissue, differences in body composition, especially in fat mass, can lead to differences in the average amount of adipokine secretion. Indeed, on average, females have higher levels of leptin and adiponectin than males [53]. Furthermore, previous studies demonstrate that sex hormones such as androgens and estrogens can directly influence the gene expression of adipokines [55–57].

The influence of sex-based differences in adipokine levels may help explain the heterogeneity by sex observed in CVD cases. For example, a sex specific influence of adipokines on the severity of coronary artery calcification (CAC) [58], coronary heart disease events [59], cIMT [60], and blood pressure progression [61] has been noted. Sex-specific positive associations between adiponectin and cardiovascular mortality (non-protective) have been observed among male patients with T2D [62] as well as associations of adipokines on CAC among female patients [63]. Thus, further studies are needed to determine the patterns of sexual dimorphisms in the effect of various adipokines on CVD and their underlying biological mechanisms.

In contrast to the widely observed protective effects of adiponectin against cardiovascular disease in previous research, this study did not observe protective associations between adiponectin and cIMT, with the exception of the metabolically healthy male subjects. Surprisingly, some strata exhibited counterintuitive positive associations between adiponectin and cIMT and inverse associations between ARI and cIMT in the female-only analyses. We note that these counterintuitive associations were particularly strengthened when tested only among females in the metabolically elevated risk group (Fig. 2 and Additional file 1: Table S1). Although several studies on cIMT reported significant inverse or null associations with adiponectin, some studies of atrial fibrillation or CV deaths reported positive associations between adiponectin and CVD-related outcomes [64, 65]. Other studies that observed positive associations between adiponectin and CMD included patients with cardiometabolic conditions such as ischemic stroke [66], T2D [67], and non-alcoholic fatty liver disease [68], which aligns with our strong evidence of effect of the metabolic health condition on the association between adipokines and cIMT. Furthermore, females in the metabolically elevated risk group displayed an inverse association between LAR and cIMT. However, the counterintuitive results of this sub-analysis were somewhat sensitive to outliers in the phenotypic distribution and should be cautiously interpreted (Additional file 1: Table S2).

The relationship between adipokines, cIMT, and metabolic health is unsurprisingly complex. For example, although controversial, increased levels of adiponectin under metabolically perturbed conditions have been coined as the ‘adiponectin paradox’ [69, 70]. Proposed factors influencing adiponectin include insulin signaling impairments, as observed among patients with Type 1 Diabetes [71], and in animal studies that found dependence of adiponectin expression on T-cadherin and glycoprotein levels [72, 73]. Adiponectin resistance may also explain adverse CVD-related outcomes despite the higher levels of adiponectin. Indeed, a previous study observed the downregulation of the adiponectin receptor despite increased levels of adiponectin expression among patients with chronic heart failure [74]. Similarly, among patients with heart failure, higher adiponectin levels were observed in comparison to healthy controls, and reduced adiponectin levels and adiponectin expression have also observed after implantation of ventricular assist devices [75]. In the context of the aforementioned research, our observed cross-sectional association between higher levels of adiponectin and higher levels of cIMT (or inverse association between ARI and cIMT) may be driven by an adiponectin resistance-like phenomenon—i.e., poor metabolic health influencing increased levels of adiponectin without original anti-atherogenic function. Of note, adiponectin levels have also been documented to be higher among females in general [76], which may possibly explain why the effect size of this relationship was particularly greater among females. In addition, the current unexpected observations may be driven by the general poorer metabolic health (e.g., higher level of insulin resistance and obesity prevalence) in the CCHC population as compared to other populations in the U.S.

Since the unexpected relationship between adiponectin and cIMT was female-specific, it merits mentioning that menopausal status may have played a role in this association. It is known that menopause and advancement of age among females are factors associated with conditions similarly exhibited in metabolic syndrome, such as changes in sex hormone levels (i.e., estrogen) and increased visceral adipose tissue [77]. Considering the average age of the CCHC female participants [mean (SD): 50.8 (13.7) years], it is plausible that the hormonal changes of menopause could influence the interplay between adipokines, metabolic health, and subsequent early atherogenesis. However, since the current study did not formally measure the menopausal status, it is hard to reveal the exact contributions of menopause to the current female-specific results.

Though emerging evidence informs our understanding of adipokines and their influence on pro-inflammatory states and consequently manifestations of atherogenesis, conflicting findings across animal and human studies indicate the need for further investigations of specific adipokines with pre-atherosclerotic states. For example, while our finding of the negative association between resistin and cIMT among metabolically healthy males (Additional file 1: Table S2) seems counterintuitive to several studies which suggest its role in pro-inflammatory pathways [78, 79] and even cardiovascular death [7], other study has also reported null associations of resistin with mean intima-media thickness [80]. As such, contradicting evidence in the literature limits our ability to conclude whether resistin may serve as a reliable marker of early atherogenesis in this population.

The current study had notable strengths. Unlike most previous studies, we assessed not only specific adipokines (adiponectin, leptin, and resistin), but also composite indices (LAR and ARI) in association with an early marker of CVD. By considering additional metrics (LAR and ARI) that have shown strong associations between adipokines and CMD, we broadened our ability to capture relationships that may suggest associations with pathophysiological states. Also, we studied an under-represented Hispanic/Latino population with known vulnerabilities to poorer health outcomes [81]. Thus, our findings contribute to a more detailed understanding of adipokines and metabolic dysfunction across diverse populations.

There were also notable limitations in the current study. Residual confounding may remain from assessing adipokine associations with cIMT among a population with poorer metabolic health states compared to the general population. Also, due to the relatively small sample sizes (especially for males), precise effect estimates and significance were somewhat sensitive to the data points in the tails of the distribution, therefore interpretations should be made cautiously. However, we carried out multiple sensitivity analyses (Additional file 1: Table S2) which all led to consistent inferences. Moreover, methodological limitations from a cross-sectional design hinder us from drawing conclusions on the observed unexpected relationships between adipokines and cIMT, particularly for adiponectin and cIMT among females. Cross-sectional analyses also limit inferences on temporality, as seen in other studies that have pinpointed antecedent metabolic states that lead to an adiponectin paradox [82]. Other important factors such as changes in body fat distribution by age and sex are associated with changes in adipokine concentrations and require assessments of these variables over time [83]. Additionally, though cIMT is an established early marker of CVD, it is crucial to assess multiple measures over time to understand patterns of how cIMT progresses into CVD within a population [84]. Furthermore, we acknowledge that stratifying participants by a subset of cardiovascular disease risk factors to denote metabolic health may not have fully captured the total metabolically unhealthy population within our study. In all, these remaining gaps demonstrate the need for longitudinal assessment of adipokines with cIMT.

## Conclusions

In conclusion, our study results add to the literature describing a complex relationship between adipokines and cIMT. We demonstrate strong sex-specific and metabolic health-dependent relationship between adipokines and cIMT among Hispanics. Our findings support the utility of the composite index of LAR as a biomarker for early atherosclerosis, especially for males and metabolically healthy individuals. We also observed an unexpected positive association between adiponectin and cIMT (and inverse association between ARI and cIMT) among females with elevated metabolic risk. Further studies exploring these relationships may lead to therapeutic targets to mitigate cardiovascular disease risk among Hispanics at metabolically elevated risk.

### Supplementary Information

**Additional file 1: Table S1.** Estimated associations between adipokines and cIMT (continuous) in participants of CCHC stratified by metabolic health and sex. **Table S2.** Sensitivity analyses for different approaches to evaluate the potential influences of upper or lower outliers on the association results. **Table S3.** The distributions of age, sex, and BMI among the whole CCHC participants and the current sub-samples.

## Data Availability

The datasets used and analyzed during the current study are available from the corresponding author on reasonable request.

## Abbreviations

ARI: Adiponectin-resistin index
BMI: Body mass index
BP: Blood pressure
CAC: Coronary artery calcification
CCHC: The Cameron County Hispanic Cohort
cIMT: Carotid-intima media thickness
CMD: Cardiometabolic diseases
CRP: C-reactive protein
CVD: Cardiovascular disease
DBP: Diastolic blood pressure
ELISA: Enzyme-linked immunosorbent assays
FBG: Fasting blood glucose
HDL-C: High-density lipoprotein cholesterol
HOMA-IR: Homeostatic Model Assessment for Insulin Resistance
IL-1β: Interleukin-1 Beta
IL-6: Interleukin-6
IL-8: Interleukin-8
LAR: Leptin-to-adiponectin ratio
LDL-C: Low-density lipoprotein cholesterol
SBP: Systolic blood pressure
SD: Standard deviation
T2D: Type 2 Diabetes
TG: Triglyceride
TNF-α: Tumor Necrosis Factor-alpha
