# Supplementary material for: Sex-specific associations between adipokine profiles and carotid-intima media thickness in the Cameron County Hispanic Cohort (CCHC)
Source: Cardiovasc Diabetol. 2023 Aug 31;22:231. doi: 10.1186/s12933-023-01968-4 (PMC10472619; doi:10.1186/s12933-023-01968-4)
Supplement: Supplementary file 1 — Additional file 1: Table S1. Estimated associations between adipokines and cIMT (continuous) in participants of CCHC stratified by metabolic health and sex. Table S2. Sensitivity analyses for different approaches to evaluate the potential influences of upper or lower outliers on the association results. Table S3. The distributions of age, sex, and BMI among the whole CCHC participants and the current sub-samples. [file 12933_2023_1968_MOESM1_ESM.docx]

**Additional File 1**

**Table S1**. Estimated associations between adipokines and cIMT (continuous) in participants of CCHC stratified by metabolic health and sex*

|  | Metabolically healthy | | | Metabolically elevated risk | | |
| --- | --- | --- | --- | --- | --- | --- |
|  | beta^a^ | SE | P | beta^a^ | SE | P |
| **Both sexes-combined** |  |  |  |  |  |  |
| Adiponectin | -0.017 | 0.010 | 0.081 | 0.008 | 0.006 | 0.144 |
| Leptin^b^ | 0.042 | 0.014 | 0.002 | -0.005 | 0.008 | 0.533 |
| Resistin | -0.015 | 0.010 | 0.147 | 0.001 | 0.005 | 0.925 |
| LAR^b^ | 0.041 | 0.009 | 4.86E-05 | -0.008 | 0.006 | 0.239 |
| ARI | 0.009 | 0.010 | 0.356 | -0.009 | 0.006 | 0.151 |
| **Female** |  |  |  |  |  |  |
| Adiponectin^b^ | -0.009 | 0.008 | 0.267 | 0.014 | 0.005 | 0.012 |
| Leptin | -0.006 | 0.013 | 0.642 | -0.005 | 0.007 | 0.499 |
| Resistin | 0.000 | 0.010 | 0.985 | 0.003 | 0.005 | 0.572 |
| LAR | 0.004 | 0.009 | 0.690 | -0.012 | 0.006 | 0.040 |
| ARI^b^ | 0.014 | 0.009 | 0.113 | -0.015 | 0.006 | 0.013 |
| **Male** |  |  |  |  |  |  |
| Adiponectin | -0.072 | 0.028 | 0.015 | -0.018 | 0.016 | 0.264 |
| Leptin^b^ | 0.188 | 0.029 | 1.04E-07 | -0.001 | 0.024 | 0.980 |
| Resistin | -0.053 | 0.021 | 0.017 | -0.004 | 0.012 | 0.731 |
| LAR^b^ | 0.127 | 0.015 | 4.70E-10 | 0.029 | 0.022 | 0.194 |
| ARI | 0.001 | 0.027 | 0.968 | 0.009 | 0.013 | 0.513 |

^*^ Liner mixed effect model was used. A kinship matrix estimated based on genotype information among samples was included as a random effect. Fixed effect covariates were age, sex, BMI, smoking, cytokines (IL-6, IL-8, TNF-a, IL-1b), glucose tolerance, BP, and lipid profile, and medication usage for T2D, hypertension, or lipid-lowering.

^a^ beta coefficients indicate the increase in cIMT levels by every 1-SD unit increase of adipokines

^b^ significant interaction by metabolic health (p for interaction < 0.1)

**Table S2**. Sensitivity analyses for different approaches to evaluate the potential influences of upper or lower outliers on the association results^**^

|  |  | **Sensitivity 1 (N=624) Winsorized (abs. values) after excl. adiponectin > 200^*^** | | | **Sensitivity 2 (N=624) Inverse-normal transformation after excl. adiponectin > 200^*^** | | | **Sensitivity 3 (N=624) No transformation after excl. adiponectin > 200^*^** | | | **Sensitivity 4 (N=631) Include all + Winsorize (5% - 95%)^*^** | | | | **Sensitivity 5 (N=631) Include all + Winsorize (abs. value) ^*^** | | | |
| --- | --- | --- | --- | --- | --- | --- | --- | --- | --- | --- | --- | --- | --- | --- | --- | --- | --- | --- |
| **All** | | beta | SE | P | beta | SE | P | beta | SE | P | beta | SE | P | beta | | SE | P |  |
| Pooled | Adiponectin | 0.001 | 0.005 | 0.795 ^¶^ | 0.003 | 0.005 | 0.604^¶^ | 0.001 | 0.005 | 0.909^¶^ | 0.001 | 0.005 | 0.879^¶,†^ | -0.001 | | 0.005 | 0.793^¶,†^ |  |
|  | Leptin | 0.008 | 0.007 | 0.230 ^¶,†^ | 0.001 | 0.007 | 0.908^¶^ | 0.008 | 0.007 | 0.215^¶,†^ | 0.003 | 0.007 | 0.664^¶,†^ | 0.008 | | 0.007 | 0.235^¶^ |  |
|  | Resistin | -0.002 | 0.005 | 0.707 | -0.003 | 0.005 | 0.552 | -0.004 | 0.005 | 0.445 | -0.002 | 0.005 | 0.703 | -0.003 | | 0.005 | 0.578 |  |
|  | LAR | 0.001 | 0.005 | 0.843 ^¶,†^ | 0.001 | 0.006 | 0.891^¶,†^ | 0.012 | 0.004 | 0.010^¶,†^ | 0.003 | 0.005 | 0.609^¶,†^ | 0.001 | | 0.005 | 0.781^¶,†^ |  |
|  | ARI | -0.005 | 0.005 | 0.376 | -0.004 | 0.005 | 0.428^¶^ | -0.001 | 0.005 | 0.821^¶^ | -0.005 | 0.005 | 0.384 | -0.003 | | 0.005 | 0.508 |  |
| Female | Adiponectin | 0.005 | 0.005 | 0.283 | 0.011 | 0.005 | 0.042^†^ | 0.004 | 0.005 | 0.375 | 0.004 | 0.005 | 0.340^†^ | 0.002 | | 0.005 | 0.659^†^ |  |
|  | Leptin | -0.001 | 0.006 | 0.850 | -0.010 | 0.007 | 0.166 | 0.000 | 0.006 | 0.965 | -0.004 | 0.006 | 0.569 | -0.001 | | 0.006 | 0.808 |  |
|  | Resistin | 0.001 | 0.005 | 0.770 | 0.002 | 0.005 | 0.704 | -0.001 | 0.005 | 0.893 | 0.000 | 0.005 | 0.923 | 0.000 | | 0.005 | 0.993 |  |
|  | LAR | -0.007 | 0.005 | 0.133 | -0.014 | 0.006 | 0.018^†^ | -0.025 | 0.017 | 0.144 | -0.008 | 0.005 | 0.098^†^ | -0.006 | | 0.004 | 0.149 |  |
|  | ARI | -0.009 | 0.005 | 0.091 | -0.010 | 0.005 | 0.056 | -0.009 | 0.005 | 0.088 | -0.009 | 0.005 | 0.087^†^ | -0.007 | | 0.005 | 0.154 |  |
| Male | Adiponectin | -0.022 | 0.014 | 0.121 | -0.019 | 0.012 | 0.100 | -0.021 | 0.014 | 0.123 | -0.022 | 0.015 | 0.138 | -0.023 | | 0.015 | 0.121 |  |
|  | Leptin | 0.046 | 0.020 | 0.020 ^†^ | 0.022 | 0.017 | 0.207^†^ | 0.050 | 0.021 | 0.017 ^†^ | 0.038 | 0.021 | 0.064 ^†^ | 0.046 | | 0.020 | 0.020^†^ |  |
|  | Resistin | -0.008 | 0.010 | 0.463 | -0.013 | 0.011 | 0.208 | -0.007 | 0.010 | 0.487 | -0.007 | 0.011 | 0.521 | -0.008 | | 0.010 | 0.463 |  |
|  | LAR | 0.092 | 0.022 | 3.58E-05 ^†^ | 0.037 | 0.013 | 0.006 | 0.017 | 0.006 | 0.005 ^†^ | 0.060 | 0.016 | 2.46E-04^†^ | 0.091 | | 0.022 | 3.58E-05^†^ |  |
|  | ARI | 0.009 | 0.012 | 0.431 | 0.013 | 0.011 | 0.255 | 0.018 | 0.011 | 0.091 | 0.009 | 0.012 | 0.448 | 0.009 | | 0.012 | 0.431 |  |
| **Metabolically Healthy** | |  |  |  |  |  |  |  |  |  |  |  |  |  | |  |  |  |
| Pooled | Adiponectin | -0.010 | 0.009 | 0.235 | -0.022 | 0.010 | 0.037 | -0.009 | 0.009 | 0.277 | -0.019 | 0.008 | 0.029 | -0.016 | | 0.008 | 0.042 |  |
|  | Leptin | 0.052 | 0.015 | 0.001 | 0.036 | 0.014 | 0.013 | 0.055 | 0.016 | 0.001 | 0.042 | 0.014 | 0.002 | 0.052 | | 0.015 | 0.001 |  |
|  | Resistin | -0.017 | 0.010 | 0.107 | -0.017 | 0.010 | 0.072 | -0.015 | 0.010 | 0.131 | -0.019 | 0.010 | 0.046 | -0.021 | | 0.009 | 0.027 |  |
|  | LAR | 0.058 | 0.014 | 5.90E-05 | 0.035 | 0.012 | 0.004 | 0.222 | 0.054 | 0.000 | 0.041 | 0.009 | 0.000 | 0.060 | | 0.014 | 2.61E-05 |  |
|  | ARI | 0.006 | 0.010 | 0.560 | 0.005 | 0.010 | 0.619 | 0.005 | 0.010 | 0.597 | 0.011 | 0.010 | 0.249 | 0.008 | | 0.010 | 0.400 |  |
| Female | Adiponectin | -0.003 | 0.007 | 0.653 | -0.016 | 0.010 | 0.103 | -0.002 | 0.007 | 0.729 | -0.015 | 0.007 | 0.031 | -0.013 | | 0.006 | 0.042 |  |
|  | Leptin | -0.004 | 0.015 | 0.790 | -0.021 | 0.015 | 0.170 | -0.004 | 0.016 | 0.790 | -0.003 | 0.014 | 0.800 | -0.001 | | 0.016 | 0.939 |  |
|  | Resistin | 0.000 | 0.011 | 0.996 | 0.001 | 0.009 | 0.895 | 0.000 | 0.013 | 0.996 | -0.011 | 0.009 | 0.229 | -0.014 | | 0.009 | 0.142 |  |
|  | LAR | 0.005 | 0.014 | 0.734 | 0.000 | 0.012 | 0.988 | 0.019 | 0.053 | 0.737 | 0.007 | 0.009 | 0.458 | 0.009 | | 0.014 | 0.507 |  |
|  | ARI | 0.011 | 0.009 | 0.204 | 0.015 | 0.009 | 0.119 | 0.011 | 0.009 | 0.221 | 0.017 | 0.009 | 0.056 | 0.015 | | 0.009 | 0.080 |  |
| Male | Adiponectin | -0.072 | 0.028 | 0.012 | -0.044 | 0.024 | 0.072 | -0.073 | 0.028 | 0.013 | -0.078 | 0.030 | 0.013 | -0.078 | | 0.030 | 0.012 |  |
|  | Leptin | 0.212 | 0.030 | 2.80E-08 | 0.112 | 0.027 | 1.66E-04 | 0.225 | 0.032 | 2.80E-08 | 0.188 | 0.029 | 1.01E-07 | 0.212 | | 0.030 | 2.80E-08 |  |
|  | Resistin | -0.047 | 0.019 | 0.018 | -0.063 | 0.021 | 0.004 | -0.028 | 0.016 | 0.096 | -0.053 | 0.021 | 0.017 | -0.047 | | 0.019 | 0.018 |  |
|  | LAR | 0.197 | 0.023 | 1.85E-10 | 0.098 | 0.024 | 1.98E-04 | 0.762 | 0.088 | 1.82E-10 | 0.126 | 0.015 | 4.55E-10 | 0.196 | | 0.023 | 1.85E-10 |  |
|  | ARI | -0.004 | 0.028 | 0.872 | -0.009 | 0.026 | 0.737 | -0.004 | 0.028 | 0.891 | 0.005 | 0.027 | 0.865 | -0.005 | | 0.028 | 0.872 |  |
| **Metabolically elevated risk** | |  |  |  |  |  |  |  |  |  |  |  |  |  | |  |  |  |
| Pooled | Adiponectin | 0.004 | 0.006 | 0.517 | 0.006 | 0.006 | 0.343 | 0.003 | 0.006 | 0.637 | 0.005 | 0.006 | 0.378 | 0.003 | | 0.006 | 0.668 |  |
|  | Leptin | 0.002 | 0.007 | 0.780 | -0.006 | 0.008 | 0.452 | 0.003 | 0.007 | 0.703 | -0.005 | 0.008 | 0.500 | 0.002 | | 0.007 | 0.827 |  |
|  | Resistin | 0.000 | 0.005 | 0.988 | -0.001 | 0.005 | 0.868 | -0.002 | 0.005 | 0.648 | 0.000 | 0.005 | 0.942 | 0.000 | | 0.005 | 0.974 |  |
|  | LAR | -0.004 | 0.006 | 0.497 | -0.004 | 0.007 | 0.563 | 0.012 | 0.005 | 0.014 | -0.007 | 0.006 | 0.280 | -0.004 | | 0.005 | 0.519 |  |
|  | ARI | -0.006 | 0.006 | 0.316 | -0.005 | 0.006 | 0.397 | -0.001 | 0.006 | 0.797 | -0.007 | 0.006 | 0.212 | -0.005 | | 0.006 | 0.375 |  |
| Female | Adiponectin | 0.008 | 0.006 | 0.155 | 0.015 | 0.006 | 0.014 | 0.007 | 0.006 | 0.228 | 0.009 | 0.005 | 0.102 | 0.006 | | 0.006 | 0.283 |  |
|  | Leptin | -0.001 | 0.007 | 0.843 | -0.011 | 0.008 | 0.194 | 0.000 | 0.007 | 0.972 | -0.005 | 0.007 | 0.450 | -0.002 | | 0.007 | 0.769 |  |
|  | Resistin | 0.003 | 0.005 | 0.598 | 0.003 | 0.006 | 0.559 | 0.000 | 0.005 | 0.965 | 0.003 | 0.005 | 0.600 | 0.003 | | 0.005 | 0.624 |  |
|  | LAR | -0.008 | 0.005 | 0.123 | -0.017 | 0.007 | 0.011 | -0.028 | 0.019 | 0.135 | -0.011 | 0.006 | 0.054 | -0.007 | | 0.005 | 0.133 |  |
|  | ARI | -0.012 | 0.006 | 0.045 | -0.014 | 0.006 | 0.026 | -0.013 | 0.006 | 0.043 | -0.013 | 0.006 | 0.030 | -0.011 | | 0.006 | 0.071 |  |
| Male | Adiponectin | -0.019 | 0.016 | 0.231 | -0.017 | 0.013 | 0.184 | -0.019 | 0.016 | 0.239 | -0.020 | 0.017 | 0.256 | -0.021 | | 0.018 | 0.231 |  |
|  | Leptin | 0.014 | 0.023 | 0.537 | 0.000 | 0.020 | 0.988 | 0.016 | 0.024 | 0.494 | -0.001 | 0.024 | 0.982 | 0.014 | | 0.023 | 0.537 |  |
|  | Resistin | -0.005 | 0.012 | 0.699 | -0.010 | 0.012 | 0.426 | -0.005 | 0.012 | 0.677 | -0.004 | 0.012 | 0.731 | -0.005 | | 0.012 | 0.699 |  |
|  | LAR | 0.056 | 0.028 | 0.044 | 0.028 | 0.015 | 0.064 | 0.017 | 0.006 | 0.007 | 0.029 | 0.022 | 0.193 | 0.056 | | 0.028 | 0.044 |  |
|  | ARI | 0.011 | 0.013 | 0.418 | 0.016 | 0.013 | 0.207 | 0.021 | 0.012 | 0.072 | 0.009 | 0.013 | 0.494 | 0.011 | | 0.013 | 0.418 |  |

*Sensitivity analysis 1: After excluding adiponectin > 200 ug/mL (N=7), winsorized extreme values by the absolute threshold for each adipokine (1 – 150 ug/mL for adiponectin, 0.5 – 100 ng/mL for leptin, and 1.28 – 100 ug/mL for resistin; LAR and ARI were derived after winsorization; Sensitivity analysis 2: Excluding adiponectin > 200 ug/mL (N=7) and inverse normal transformation for each adipokine; Sensitivity analysis 3: Excluding adiponectin > 200 ug/mL (N=7) and no further transformation was made; Sensitivity analysis 4: Included all complete cases and winsorized by relative threshold (5% - 95% for each adipokine); Sensitivity analysis 5: Included all complete cases and winsorized extreme values by the absolute threshold for each adipokine.

** All results were from fully-adjusted model (Model 5)

^¶^ Significant interaction by sex

^†^ Significant interaction by metabolic health status

**Table S3**. The distributions of age, sex, and BMI among the whole CCHC participants and the current sub-samples

|  | **Current study** | **Total CCHC samples at baseline** |
| --- | --- | --- |
| N | 624 | 5020 |
| Age | 50.2 (14.4) | 42.6 (17.8)^*^ |
| Sex |  |  |
| Male | 182 (29.1%) | 1858 (37.0%) |
| Female | 442 (70.8%) | 3162 (63.0%) |
| BMI | 30.7 (6.03) | 30.5 (8.83)^**^ |

^*^ 3 missings

^**^ 21 missings
